# Major approaches in early diagnostics of common variable immunodeficiency in adults in Moscow

**DOI:** 10.12688/f1000research.1-46.v1

**Published:** 2012-11-09

**Authors:** Alexander V Karaulov, Irina V Sidorenko, Anna S Kapustina

**Affiliations:** 1 I.M. Sechenov First Moscow State Medical University, Moscow, 119991, Russian Federation

## Abstract

Common variable immunodeficiency (CVID) is a primary immunological disease characterized predominantly by hypogammaglobulinemia. The main clinical manifestations are severe recurrent infections that often lead to structural damage of affected organs. The early start of adequate intravenous immunoglobulin therapy has significantly improved the prognosis of this serious disorder. Patients with CVID are also predisposed to autoimmune and lymphoproliferative complications. This article deals with the features of this primary immunodeficiency in adults. Clinical manifestations, immunological features and treatment concepts were gathered during 21 years of observation of such patients in Moscow. The authors suggest early predictive clinical signs of CVID in adults.

## Background

Common variable immunodeficiency is a primary immunodeficiency (PID) characterized by variable age of onset of symptoms, recurrent severe bacterial infections, increased incidence of autoimmune disorders and malignancy ^1– 3^. CVID is the most frequent symptomatic primary immunodeficiency in adults ^1^. CVID is usually diagnosed in patients presenting with hypogammaglobulinemia and a clinical history of recurrent and severe infections, mostly affecting the respiratory tract ^1– 3^. To confirm a diagnosis of CVID, it is important to exclude other primary antibody deficiency syndromes and secondary causes of hypogammaglobulinemia ^1^.

## Materials and methods

We observed 57 patients with CVID in 1990–2011. Written informed consent for publication of clinical details was obtained from all patients or their next of kin.

We conducted a comprehensive examination, treatment and, quality-of-life study of 27 males and 30 females aged from 18 to 74 (mean: 39±1, 95 years). The medical history was collected from all patients (emphasis being on the manifestation of first symptoms, mainly affected organs and systems), as was family history, the dynamics of clinical symptoms and, the effectiveness of the therapy. All patients were physically examined, and blood and urine tests were also conducted. We also used X-rays and computed tomography of the chest and sinuses, esophagogastroscopy, colonoscopy (indication), ultrasonography of the abdomen, kidneys and, thymus, research conducted on electrocardiogram (ECG) and, respiratory function. Patients were examined by an ear, nose and throat (ENT) specialist, gastroenterologist, pulmonologist, hematologist and, a rheumatologist.

Immunological testing included the study of the following parameters of the immune system: the total number of leukocytes and lymphocytes, the percentage and absolute number of T-lymphocytes (CD3 ^+^-cells) and subpopulations of T-lymphocytes (CD4 ^+^-cells, CD8 ^+^-cells), natural killer (NK)-cells (CD16 ^+^-cells) and, B-lymphocytes (CD19 ^+^-cells) in peripheral blood. Levels of immunoglobulin classes A, M, G and, E in serum were determined by nephelometry. Immunophenotyping of peripheral blood lymphocytes was measured by cytometry using monoclonal antibodies. Autoantibodies were measured by indirect immunofluorescence. For detection of red-cell antibodies we used Coombs' reaction. The phagocytic link of immunity was investigated by determining the absolute and percentage content of neutrophils and monocytes, the absorptive activity of leukocytes, phagocyte chemiluminescence and bactericidal activity of leukocytes.

Assessment of treatment efficiency was carried out by analyzing the dynamics of clinical and immunological parameters, measuring the duration of remission of the underlying disease and, studying the patient's of quality of life. Monitoring of serum immunoglobulin G (IgG) was carried out before the infusion of intravenous immunoglobulin (IVIG) to evaluate the achievement of adequate levels of IgG (not less than 5.0 g/l).

Analysis of the incidence of infectious complications and receiving antibiotics was carried out using the questionnaire "Total well-being: the emergence of infectious complications" filled prior to the IVIG infusion and, on the 2nd and 10th day after the transfusion medicine.

Questionnaire: total well-being - the emergence of infectious complications Questionnaire used to analyze the incidence of infectious complications and antibiotic administration related to intravenous immunoglobulin therapy (IVIG).

Assessment of the safety of IVIG was performed on the basis of data on tolerability, and analysis of the patient’s vital signs and markers of renal function. Before each infusion of IVIG, as well as on the 2 ^nd^ and 10 ^th^ day after the transfusion the patients completed a questionnaire "Total well-being: the emergence of reactions, possibly related to the introduction of IVIG" pointing out any reactions that had arisen since the last replacement therapy.

Questionnaire: total well-being - the emergence of reactions, possibly related to the introduction of IVIG Questionnaire used to identify any negative reactions arising since the last intravenous immunoglobulin therapy (IVIG).

In the case of a new drug being prescribed to the patient, the tolerability of IVIG was assessed during the first infusion by monitoring blood pressure, heart rate, body temperature, and studying the markers of renal function. Biochemical blood tests were conducted with a focus on the concentration of urea, electrolytes and creatinine before the first infusion, and on the second and fifth days after infusion.

To study patient quality of life, we used the Russian version of the general health questionnaire MOS SF-36 (36-item Measures of Sickness Short-Form Health Survey). The results were displayed on a scale from 0 to 100 points. A high rate showed a good quality of life of the respondent, a low showed a bad quality of life.

On the basis of this study, we worked out early predictive clinical signs of CVID for primary care.

## Results

In the observed group, duration of illness ranged from 3 to 62 years (mean 23.6±1,18 years). The first symptoms appeared in 28 patients (49.1%) before 15 years and in 29 patients (50.9%) over 15 years. The disease started in 10 patients in the first decade of life, in 25 patients in the second decade of life, in 13 patients in the third decade of life, in 4 patients at 30 to 40 years, and in 5 patients over 40 years.

Family history was burdened in 5 patients (8.8%): early death of children (with probable immunodeficiency) from infectious complications took place in 2 families; the brother (with unspecified immunodeficiency) of one patient died of toxoplasmosis; the son of one patient and the mother and son of the other patient have primary immunodeficiency – selective IgA-deficiency.

The main clinical manifestations were bacterial infections of the respiratory system; this occurred in 53 patients (93%). CVID began with recurrent purulent sinusitis in 57.9% of cases, ear infections in 26.3%, bronchitis in 54.4% and, pneumonia in 56.1%. In 4 patients (7%), the first clinical manifestation of CVID was damage to the gastrointestinal tract (enterocolitis and malabsorption syndrome). In 6 patients (10.5%), disease also began with autoimmune cytopenias: hemolytic anemia in 2, neutropenia in 1 and, thrombocytopenia in 3 patients ( Table 1).

**Table 1.** Early clinical signs of CVID.

Early clinical signs	Number of patients	Percentages
Purulent sinusitis	33	57.9
Ear infections	15	26.3
Purulent bronchitis	31	54.4
Recurrent pneumonia	32	56.1
Enterocolitis	4	7.0
Autoimmune cytopenias	6	10.5

Before the diagnosis of PID and the use of replacement IVIG therapy, foci of chronic infection of the respiratory system formed in 54 patients (94.7%): chronic bronchitis 94.7%, chronic sinusitis 75.4% and, chronic otitis media 42.1%. Recurrent diarrhea and weight loss were observed in 47.4% patients. Septicemia, meningitis and, peritonitis were found in 15.8% cases. Severe clinical presentations required re-hospitalization including in intensive care units. Because of late assignment of IVIG therapy, chronic infection foci of the respiratory system formed in 94.7% of patients: bronchiectasis – 36.8%, local fibrosis – 50.9% (lobectomy was performed in 2 patients), chronic sinusitis – 75.4% ( Table 2). Despite severe manifestations, a delay in diagnosis of CVID and consultation by immunologist was from 2 to 45 years (mean: 14.5 years).

**Table 2.** Main clinical manifestations of CVID.

Manifestations	Number of patients	Percentages
Chronic bronchitis	54	94.7
Bronchiectasis	21	36.8
Local fibrosis	29	50.9
Chronic sinusitis	43	75.4
Recurrent herpes infection	27	47.4
Generalized infections	9	15.8
Malabsorption syndrome	27	47.4
Autoimmune diseases	14	24.6
Malignancies	9	15.8

In 24.6% of cases, autoimmune diseases were observed, including hemolytic anemia, thrombocytopenia, neutropenia (also in the manifestation of the disease), and nephritis, hepatitis, and scleroderma. A total of 47.4% of patients had recurrent herpes infection and, 47.4% of cases had evidence of non-malignant lymphoproliferation: an increase of peripheral lymph nodes, intestinal lymphoid hyperplasia and, splenomegaly, which occured separately or combined. In total, 15.8% of patients had malignancies: cancer of the stomach, colon, breast, ovarian, T-cell lymphoma, chronic lymphocytic leukemia and, multiple myeloma ( Table 2). 40.4% of patients had manifestations of arthritis involving the knee, ankle, elbow and, wrist joints.

The initial investigation of immune status revealed reduction in the total IgG, IgA and, IgM levels in 89.5% of patients. In 2 cases, normal values of IgA were detected; in 4 cases normal values of IgM were shown. In all patients, IgG levels were below 4 g/l and in 36.8% only trace concentrations were identified ( Table 3).

**Table 3.** Levels of immunoglobulin classes in patients with CVID before regular IVIG replacement therapy.

Levels of immunoglobulin classes	Number of patients	Percentages
Reduction in the total IgG, IgA and, IgM levels	51	89.5
IgG < 4.0 g/l, of them IgG < 1.0 g/l	57 21	100.0 36.8
Normal values of IgA	2	3.5
Normal values of IgM	4	7.0

The content of B-lymphocytes (CD19 +) was within normal limits in 41 patients (71.9%), increased up to 21.1–30.9% (0.58–0.83×10 ^9^/L) in 6 patients (10.5%) and, reduced to 1.9–4.9% (0.01–0.1×10 ^9^/L) in 10 patients (7 women and 3 men). In 29 patients (50.9%) CD4+- cells were reduced to 16.4–24.7% (0.14–0.3×10 ^9^/L). A total of 22 patients (38.6%) had elevated levels of CD8 ^+^-cells to 37.2–65.0% (0.77–1.82×10 ^9^/L). 56.1% of cases (32 patients) showed a reduction of NK-cells to 1.8–4.4% (0.01–0.09×10 ^9^/L).

All patients received combined therapy with mandatory regular IVIG infusion ^4, 5^. At the beginning of treatment, the patients received IVIG preparations at a dose of 0.3 g/kg body weight weekly for 1.5–2 months. After reaching the required level of IgG (5.0 g/l), patients received regular IVIG replacement therapy at a dose of 0.4–0.5 g/kg ^6, 7^. The introduction of a drug was conducted once every 4 weeks (an average of 1 per 30.5±1.5 days) intravenously.

IVIG dose adjustment was carried out, taking into account the response to therapy, monitoring of foci of chronic infection, the presence of autoimmune disease and, malabsorption syndrome, and was conducted under the mandatory supervision of IgG levels in the serum, which was 5.0–8.0 g/l (mean 6.8±0.29 g/l) in all cases except for 4 patients, who did not comply with IVIG regimen, where it was 3.5–4.3 g/l.

Side effects of IVIG therapy occurred in 16 (41%) patients. In total, 4 (10%) patients had systemic reactions within 20 minutes after the start of IVIG – lowering of blood pressure to 60/30 mm-Hg, bronchospasm and, vomiting. In 12 (31%) patients, after transfusion body temperature was recorded to rise up to 37.3–37.9°C. Additionally, chills, headache, dizziness and general weakness were observed. These symptoms characterize a reaction to the introduction of a large dose of protein, which is not an indication to stop administering the drug. Since IVIG is vital, individual selection of drugs was conducted, if necessary, patients received premedication with corticosteroids, antihistamines or inhibitors of prostaglandins, which were pre-introduced (20–30 minutes prior to transfusion of IVIG) to avoid possible adverse reactions.

Replacement therapy in most cases is insufficient to control the foci of chronic infection, so in parallel with the introduction of IVIG, 40 (82%) of patients received broad-spectrum antibiotics. Due to this treatment the frequency of exacerbations of chronic foci of infection was significantly reduced. Four patients received acyclovir permanently because of the continuous recurrence of herpes infection. Immunosuppressive therapy was conducted in 7 patients with autoimmune thrombocytopenia and anemia. Hematopoietic growth factors were used in 5 patients with neutropenia, and 2 patients received ongoing treatment with growth factors. Due to the complex treatment, exacerbation of chronic bronchitis, sinusitis, otitis media and, herpes occured less often and less severely. The symptoms of malabsorption and reactive arthritis regressed, autoimmune cytopenia remission was achieved in all patients.

The clinical characteristics of 57 patients with CVID The data file “The clinical characteristics of 57 patients with CVID” contains the significant features of common variable immunodeficiency in adults. The early clinical signs and main clinical manifestations of this primary immunodeficiency were gathered during 21 years of observation of such patients in Moscow

## Conclusions

During the first 5 years of observation in our clinic, in spite of regular IVIG therapy, 21% patients died due to late diagnosis of CVID, with very severe clinical presentations and complications. Therefore, we worked out early predictive clinical signs of CVID in adults on the basis of the recommendations of European Society for Immunodeficiencies (ESID):

1) ≥ 4 bacterial respiratory tract infections (otitis, sinusitis, bronchitis, pneumonia) during 1 year;

2) ≥ 2 radiologically confirmed pneumonias during 3 years;

3) Recurrent or chronic bacterial respiratory tract infections combined with recurrent diarrhea;

4) Bacterial infections of respiratory tract and/or recurrent diarrhea combined with severe generalized infections (septicemia, meningitis, osteomyelitis);

5) Any of the above criteria combined with autoimmune diseases, especially autoimmune hemocytopenias (hemolytic anemia, thrombocytopenia, neutropenia).

6) Chronic or recurrent bacterial infections of respiratory tract and/or malabsorption syndrome combined with malignancies, with bacterial infections preceding malignancies.

Thus, increasing awareness of PID among physicians is needed to reduce the number of undetected cases and to decrease the diagnostic delay. The developed approach and education of GPs has improved the diagnostics of predominantly antibody deficiencies in adults in Moscow.

## Consent

Written informed consent for publication of clinical details was obtained from all patients or their next of kin.
