# Supplementary material for: Major approaches in early diagnostics of common variable immunodeficiency in adults in Moscow
Source: F1000Res. 2012 Nov 9;1:46. [Version 1] doi: 10.12688/f1000research.1-46.v1 (PMC3782364; doi:10.12688/f1000research.1-46.v1)
Supplement: Questionnaire: total well-being - the emergence of infectious complications — Questionnaire used to analyze the incidence of infectious complications and antibiotic administration related to intravenous immunoglobulin therapy (IVIG). [file f1000research-1-215-s0001.tgz › Total_well_being.pdf]

QUESTIONAIRE

Total well-being: the emergence of infectious complications

What are your complaints now?

- ☐ dry cough
- ☐ cough with light sputum
- ☐ cough with yellow sputum
- ☐ cough with green sputum
- ☐ wheezing
- ☐ shortness of breath
- ☐ throat ache
- ☐ pain in the projection of the paranasal sinuses
- ☐ nasal congestion
- ☐ watery discharge from the nose
- ☐ yellow discharge from the nose
- ☐ green discharge from the nose
- ☐ diarrhea
- ☐ abdominal pain
- ☐ redness of the eyes
- ☐ light discharge from the eyes
- ☐ yellowdischarge from the eyes
- ☐ green discharge from the eyes
- ☐ ear pain
- ☐ discharge from the ear canal
- ☐ fever
- ☐ herpes
- ☐ arthralgia
- ☐ other (specify the complaint):
- ☐ regimen of antibiotics:

Date________________
