# Supplementary material for: Major approaches in early diagnostics of common variable immunodeficiency in adults in Moscow
Source: F1000Res. 2012 Nov 9;1:46. [Version 1] doi: 10.12688/f1000research.1-46.v1 (PMC3782364; doi:10.12688/f1000research.1-46.v1)
Supplement: Questionnaire: total well-being - the emergence of reactions, possibly related to the introduction of IVIG — Questionnaire used to identify any negative reactions arising since the last intravenous immunoglobulin therapy (IVIG). [file f1000research-1-215-s0000.tgz › Total_well_being_the_emergence_of_reactions_possibly_related_to_the_introduction_of_IVIG.pdf]

QUESTIONAIRE

Total well-being: the emergence of reactions, possibly related to the introduction of IVIG

In the process of administration of intravenous immunoglobulin or after transfusion did you have the following side effects?

☐ headache

☐ dizziness

☐ backache

☐ abdominal pain

☐ nausea

☐ vomiting

☐ fever

☐ chill

☐ myalgia

☐ skin rash

☐ feeling short of breath

☐ wheezing

☐ reduction in blood pressure

☐ increase in blood pressure

☐ weakness

☐ other (specify the type of reaction):

Date________________
