# Supplementary material for: Major approaches in early diagnostics of common variable immunodeficiency in adults in Moscow
Source: F1000Res. 2012 Nov 9;1:46. [Version 1] doi: 10.12688/f1000research.1-46.v1 (PMC3782364; doi:10.12688/f1000research.1-46.v1)
Supplement: Peer review history

# Peer review: Referee response for version 1

**Published:** 2012-11-21

**Authors:** Francisco A Bonilla

**Affiliations:** 1 Department of Allergy/Immunology, Children's Hospital Boston, Boston, MA, USA

The authors only identified 57 patients with common variable immunodeficiency (CVID) over 21 years. This must represent only a fraction of the total number for such a large metropolitan area over such a long period of time. I suspect that many patients have gone undiagnosed and this leads me to question the general applicability of some of the authors’ findings.

Furthermore, the authors do not include their case definition of CVID. There are many immunodeficiencies associated with hypogammaglobulinemia that would not be classified as CVID. The authors state that they studied quality of life in these patients, but they do not report any of that data here. The humoral and cellular immunologic data should be presented in a table with mean, standard deviation, median and range together with the authors’ normal ranges. The authors speak very generally about a variety of therapeutic measures applied in addition to IgG but doesn’t provide any quantitative (or often even qualitative) report of the response (or not) to these therapies. The mortality in these patents seems high. How does it compare with published assessment of mortality in CVID? Finally, the authors do not provide any evidence to support their conclusion that “The developed approach and education of GPs has improved the diagnostics of predominantly antibody deficiencies in adults in Moscow”.

I have read this submission. I believe that I have an appropriate level of expertise to state that I do not consider it to be of an acceptable scientific standard, for reasons outlined above.

# Response

**Published:** 2012-12-05

**Authors:** Alexander Karaulov

**Affiliations:** I.M. Sechenov First Moscow State Medical University, Russian Federation

The article deals with data on the analysis of characteristics of adult patients with CVID in Moscow. The article reflects the work done during 20 years to improve the diagnosis of CVID and introduction of IVIG in Moscow.

The diagnosis of CVID was verified on the basis of the typical clinical presentations and immunological deviations. CVID was diagnosed according to the criteria of this form of PID developed by ESID and PAGID. Other causes of hypogammaglobulinemia were excluded in these patients. For example, genetic typing was conducted to exclude X-linked agammaglobulinemia.

The article focuses on the clinical manifestations of CVID, their characteristics, variety, the defeat of many organs and systems. The purpose of this scientific article is to draw attention of GPs to the problem of CVID. Due to awareness of the early clinical signs of CVID, GPs send patients to an immunologist. Thereby, a delay in diagnosis of CVID decreases. During past 10 years a delay in diagnosis of CVID in Moscow decreased from 14.5 to 6.4 years; due to early start of IVIG therapy quality of life has increased significantly – no operations on organs of respiratory tract in recent years.

At the beginning of the formation of Moscow registry of adult patients with CVID there were only 4 alive patients over 18 years. Over the years of observation a number of adult patients has increased by more than 10 times. This is due to improvement of diagnosis of CVID and increase GPs, knowledge.

More detailed characteristics of immunologic abnormalities in patients with CVID, the assessment of quality of life, more detailed description of the treatment strategy and the effectiveness of drugs will be presented in the following publications.

# Peer review: Referee response for version 1

**Published:** 2012-11-16

**Authors:** Anete Grumach

**Affiliations:** 1 Departments of Pediatrics and Dermatology, University of São Paulo Medical School, São Paulo, Brazil

The manuscript describes a Russian experience with common variable immunodeficiency (CVID) patients. Although it is a descriptive report, there is restricted data published about Russian groups presenting primary immunodeficiency (PID) and this is a report from a State University. It helps provide an indication and raises awareness for the physicians with regards to PID diagnosis considering that 51 CVID cases were collected in 21 years of observation. No molecular studies were performed.

I have read this submission. I believe that I have an appropriate level of expertise to confirm that it is of an acceptable scientific standard.

# Peer review: Referee response for version 1

**Published:** 2012-11-09

**Authors:** Cem Akin

**Affiliations:** 1 Division of Rheumatology, Immunology and Allergy, Harvard Medical School, Brigham & Women's Hospital, Boston, MA, USA

I have read this submission. I believe that I have an appropriate level of expertise to confirm that it is of an acceptable scientific standard.
